# An Augmented Neural Network for Sentiment Analysis Using Grammar

**DOI:** 10.3389/fnbot.2022.897402

**Published:** 2022-07-01

**Authors:** Baohua Zhang, Huaping Zhang, Jianyun Shang, Jiahao Cai

**Affiliations:** School of Computer Science and Technology, Beijing Institute of Technology, Beijing, China

**Keywords:** sentiment analysis, multi-channel CNN, grammar, augmentation, morphological

## Abstract

Understanding human sentiment from their expressions is very important in human-robot interaction. But deep learning models are hard to represent grammatical changes for natural language processing (NLP), especially for sentimental analysis, which influence the robot's judgment of sentiment. This paper proposed a novel sentimental analysis model named MoLeSy, which is an augmentation of neural networks incorporating morphological, lexical, and syntactic knowledge. This model is constructed from three concurrently processed classical neural networks, in which output vectors are concatenated and reduced with a single dense neural network layer. The models used in the three grammatical channels are convolutional neural networks (CNNs), long short-term memory (LSTM) networks, and fully connected dense neural networks. The corresponding output in the three channels is morphological, lexical, and syntactic results, respectively. Experiments are conducted on four different sentimental analysis corpuses, namely, hotel, NLPCC2014, Douban movie reviews dataset, and Weibo. MoLeSy can achieve the best performance over previous state-of-art models. It indicated that morphological, lexical, and syntactic grammar can augment the neural networks for sentimental analysis.

## Introduction

In the human-computer dialog, it is very important to recognize and understand the human sentiment, which can make human-computer interaction more intuitive, genuine, and natural. In the same scenario, the robot needs to choose appropriate feedback methods according to the different emotions of users. Sentimental analysis algorithms can give robots the ability to understand human emotions, but real interaction scenarios have high requirements for speed and accuracy, and existing methods are not effective.

Sentimental analysis is one of the traditional tasks in the field of natural language processing (NLP). In recent years, in large part due to increased internet access, a massive corpus of user opinion data is now available. The amount of these data on social media sites increases almost exponentially every day (Cheng et al., 2017). Using sentiment analysis technology to mine these data quickly is a hotspot of modern computer science research and software development.

More researchers have begun to do sentiment analysis using deep learning methods, such as bi-directional long short-term memory (BiLSTM), TextCNN, recurrent convolutional neural networks (RCNNs) (Kim, 2014; Zhou et al., 2016; Yang et al., 2019; Yuan et al., 2020), etc. These neural network models can automatically learn the features of the text and have significantly increased classification accuracy over former models; however, by directly mapping the input text to a vector space first and then learning the characteristics of different types of text through the optimization of a matrix used by the neural network model have adequately satisfactory results, but do not emphasize the influence of grammatical rules (Cepukenas et al., 2015) of the input text. Our research shows that using the sentimental feature of the lexical which is the sentiment word of the whole sentence, in addition to the sentence features, as the input of the model can improve the model's ability to acquire the sentiment features of the sentence. Our experiments on Chinese texts, sentence structures, sentence patterns, punctuation, and the variety of conjunctive words contained in the sentence (we called them collectively syntactic rules) can collectively have positive impacts on the process of calculating the sentimental value of the input sentences. For example, the following sentence can be considered: “Although the weather is bad today and I missed the car when I went out, I still felt very happy today (虽然天气不好且出门误了车, 但是我今天玩的很开心).” It can be seen that the sentiment category of the first-half sentence is negative and the second-half sentence is positive, but the total sentimental value of the sentence is positive. The sentiment of the first-half sentence does not affect the sentiment of the whole sentence. This part of the information will be lost if only the text features are used as input. Our experiments hypothesized that using grammatical features of the input sentence as another input of the neural network could obtain more comprehensive feature extraction and classification accuracy.

**Figure 1:**
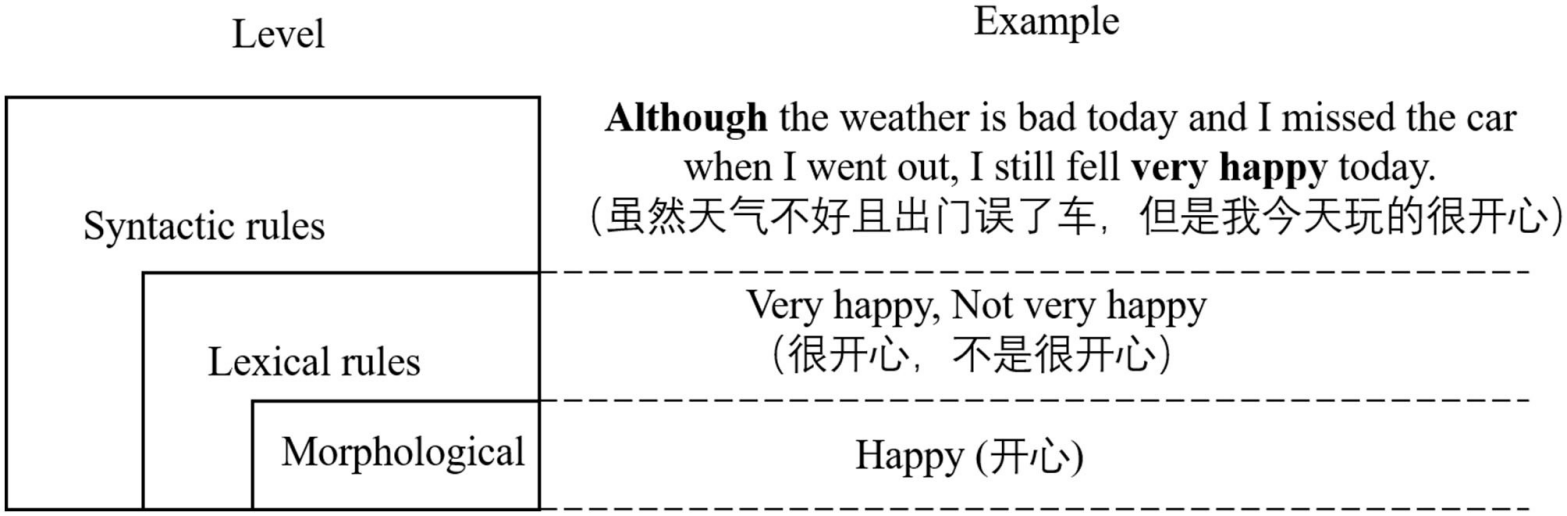
The relation of the three levels.

We divided the grammar rules into three levels, namely, morphological level, lexical rules (Zhang et al., 2015), and syntactic rules. Figure 1 shows the relations of different levels. The morphological level is the bottom layer, which is the word input commonly used in sentimental analysis models. Complex sentiment word grammar rules are at the upper level of the morphological level, which are the rules about calculating the weight of complex sentimental words according to the different combinations of the simple sentiment words, negators, and degree adverbs. The syntactic rules are at the top level, which is explained in the “Syntactic rules level” section.

Based on the research of the neural network model and the three different grammar rule levels, this paper proposed MoLeSy, which takes the morphological level, lexical rule features, and syntactic rule features all as input in a parallel fashion. First, those inputs are simultaneously input to three independent neural networks and then merged into a single feature to get the input to the final dense neural network layer. The experiment results showed that MoLeSy can extract more comprehensive text features and have more accurate output in sentiment analysis in comparison to previously existing models. Thus, the three main contributions of this study are as follows:

(1) The proposal of MoLeSy, an augmented neural network for sentiment analysis using grammar, which can obtain more comprehensive text features, and the accuracy of the inference of sentiment is higher than other models.
(2) The novel proposal is to use morphological level, lexical rules, and syntactic rules as separate features and input them into different models for feature extraction to obtain other text features of sentences from the perspective of grammar rules.
(3) The memorization of the grammar rules in Chinese, resulting in the research and development of a vector mapping method, and the construction of a feature extractor that directly extracts corresponding grammar features from sentences and converts them into feature vectors.

## Related Work

Sentiment analysis is the main technical means for analyzing user attitudes and opinions. The current sentiment analysis techniques mainly include lexicon-based methods, machine learning algorithms, and deep learning models. The methods based on a sentiment lexicon need to construct or collect the sentiment lexicon first, then perform syntactic analysis on the sentence or directly match the sentiment word, and finally, get the sentiment score of the sentence by accumulating the sentiment weight of the sentiment word. This method is simple but yields unsatisfactory results. The machine learning algorithms of sentiment analysis are often regarded as a classification of supervised machine learning, and the most commonly used methods are mainly maximum entropy, naive Bayes, and support vector machine (SVM). The machine learning method has a better classification result than the sentiment lexicon method, but also relies on manual feature selection and does not have satisfactory scalability.

Deep learning was first applied to the field of NLP by Collobert (Collobert et al., 2011) in 2011 to solve problems such as part-of-speech tagging. In 2014, Kim (2014) first used a convolutional neural network (CNN) (TextCNN) for text classification and achieved significantly improved classification results. Then, Kalchbrenner et al. (2014) proposed a wide convolution model and chose to use k-max pooling instead of the traditional CNN maximum pooling to retain more features. Zhang and Wallace (2017) compared the effects of different hyperparameters on the performance and stability of the CNN model structure through many repeated experiments. Gao et al. (2014) and Shen et al. (2014) introduced how to use CNN to learn to represent sentences into semantic structures. Zhang et al. (2016) proposed reliance on sensitive CNN and constructed a hierarchical text representation by processing pretrained word embeddings. CNNs are often used to capture local features, while recurrent neural networks (RNNs) can retain memory information due to their own feedback loop structure and have been applied in time series models (Cho et al., 2014). But, RNNs have shortcomings, as it has satisfactory results only on short input text, and when the length of the text increases, RNNs tend to exhibit gradient disappearance and gradient explosions (Li and Qian, 2016). LSTMs (Gers et al., 2000) and gated recurrent units (GRUs) (Ma et al., 2018) introduce the gate mechanism based on the traditional RNN, yielding improved even further improved results. Socher et al. (2013) proposed the tree-LSTM model, which can produce more text features. Tran et al. (2016) introduced an additional external memory unit based on LSTM to improve the model's ability to process historical information. However, due to the addition of a large number of parameters, the accuracy of this model was not improved much by this. Chen et al. (2017) proposed BiLSTMs, which both pre-order memory and post-order information, yielding better results. Wang et al. (2016a) established the AE-LSTM and ATAE-LSTM neural network models and finally obtained the sentimental classification of the aspect by modeling the LSTM, combining the hidden state of the text and the aspect information to generate the attention vector after the context modeling. But LSTM has shortcomings too. Although it can obtain the contextual semantic information of the text, it lacks the acquisition of the local information of the text. It can be seen from this that the simple model itself has certain limitations on text data processing due to its structure.

To use the advantages of RNN and CNN models at the same time, researchers began to merge the two types of models for text sentimental analysis. Wang et al. (2016b) constructed a fusion model by fusing a single-layer RNN and a single layer CNN and conducted experiments on a short text data set, and the results proved that the fusion model was outperforming the single model effect. Xu et al. (2018) proposed a CNN with dual attention vectors. Zhou et al. (2000) proposed a model that combines CNNs and LSTMs serially. The text is first convolved through CNN and then input into LSTM to obtain features. Yoon and Kim (2017) proposed a multichannel CNN_BiLSTM model combined with a dictionary. First, the text and dictionary are simultaneously input into the multichannel CNN to obtain the text features, and then the features are combined and then input into a BiLSTM for classification. Vo et al. (2017) suggested a parallel multichannel CNN and LSTM model. After embedding the text, it is input into the multichannel CNN and LSTM at the same time, and then the text features output by the two models are merged. After merging, they are input into the fully connected neural network. The experiment shows that the effect of this model on the separation of emotions in Vietnamese is better than that of a single model.

With the development of deep learning, an architecture combining multiple models has gradually become the mainstream of sentimental analysis methods. Wang et al. (2016c) proposed a regional CNN LSTM model. Yang et al. (2020) proposed a typical parallel two-layer network structure with multichannel CNN and BiGRU, which combines the features obtained by the two models when training. Li et al. (2020a) used a parallel structure to combine a BiLSTM and CNN. The difference is that they proposed a method of filling with emotional words, which can alleviate the problem of gradient disappearance. Li et al. (2020b) did not use a CNN model, but did use three BiLSTMs in parallel, and constructed the input of the three models by concatenating the part-of-speech vector, position vector, and dependent grammar vector with the text vector. Then, the attention mechanism is used to splice the three features. In this way, some grammar rules can be introduced to improve the accuracy of the model.

Yang et al. (2020) used the serial structure to combine CNNs and BiGRUs and added the attention mechanism to the last layer. Usama et al. (2020) used the serial method to combine an RNN and CNN, and the attention mechanism is added between the two models. Lin et al. (2020) proposed a comparison enhanced Bi-LSTM with multi-head attention (CE-B-MHA). Basiri et al. (2021) proposed a new model that not only has a parallel structure but also a serial structure. This model first combines a BiLSTM and BiGRU in parallel and then uses a CNN to perform convolution operations to extract local features and reduce feature dimensions. This architecture of combining multiple models has advantages over single independent models, but only Li et al. (2020a) incorporated the use of a few grammar rulers, while others ignore the influence of grammar entirely. Finally, Cheng et al. (2020) mentioned in their conclusion that it is necessary to consider integrating the syntactic structure features of traditional methods into the deep learning model, which is the main research area of this paper.

Although the existing research methods have begun to use the method of combining multiple models for sentimental analysis tasks, the current methods are still mainly based on input sentences, and a small number of models also use sentimental dictionaries as input. However, few researchers pay attention to sentimental words and grammar features that play an important role in text sentiment. The research included in this paper indicates that when the sentimental analysis is performed, more text features can be obtained as text input at the same time, and the classification accuracy is improved.

## The MoLeSy

**Figure 2:**
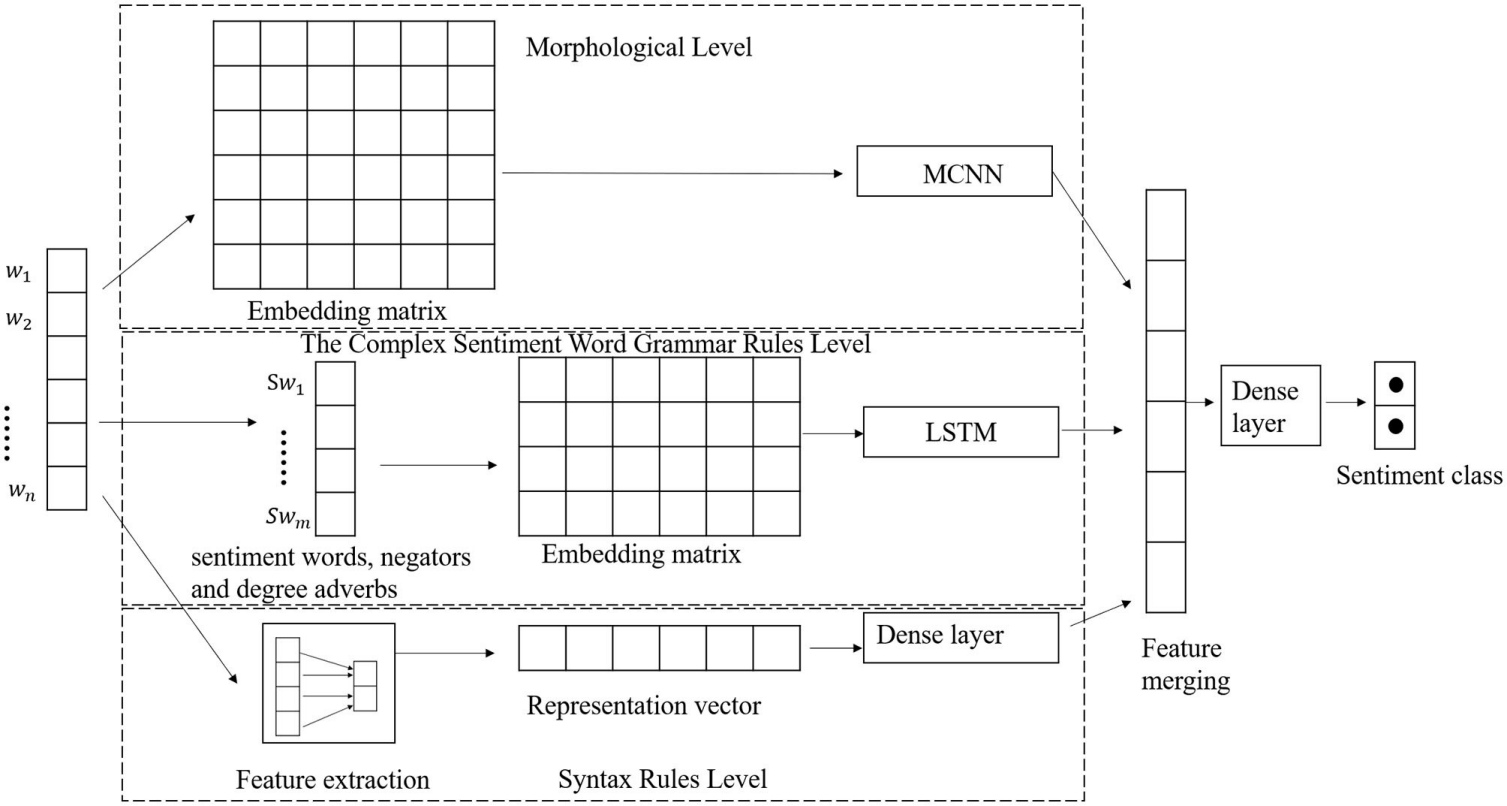
The architecture of MoLeSy.

As shown in Figure 2, our model also includes three parts. The morphological level (refer to the “Morphological level” section for details), the lexical rules level (refer to the “Lexical rules level” section for details), and the syntactic rule level (refer to the “Syntactic rules level” section for details). In this model, the input of the multichannel CNN (MCNN) model is the word embedding vector matrix of the entire sentence. The text feature will be extracted from the multichannel CNN. The input of LSTM only includes the sentimental words, negators, and the degree adverbs, which have an important impact on the sentiment of the sentence. Since these words are more representative of the sentiment of the sentence, other factors are removed. We used LSTMs to extract the features of these words. The feature extractor mainly extracts the grammar features in sentences and converts them into feature vectors as the input of the fully connected layer. Then, we can get the feature extracted from the fully connected layer. Finally, the features obtained from the three models are merged and then used as input to the fully connected neural network to combine all the features of the text and classify them.

### Morphological Level

**Figure 3:**
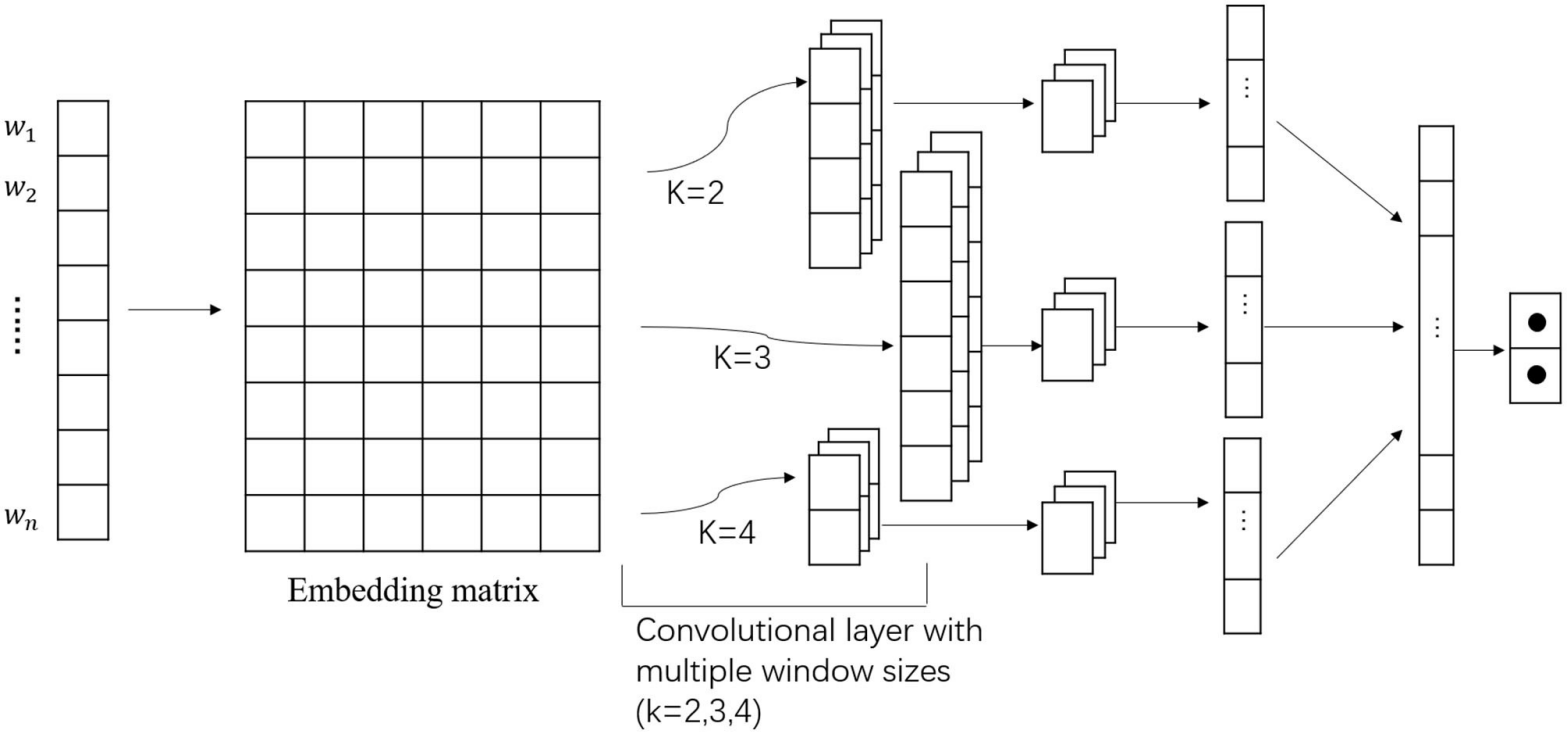
The architecture of multichannel convolutional neural network (CNN).

We used multichannel CNN to extract the morphological features from the text. Figure 3 shows the details of our multichannel CNN. It is mainly composed of a convolutional layer, a pooling layer, and a fully connected layer. The convolutional layer is used to extract the features of the input data. It contains multiple convolution kernels. After the feature extraction of the convolutional layer, the output feature map will be passed to the pooling layer for feature selection and information filtering. The fully connected layer is equivalent to the hidden layer of the traditional feed-forward neural network and is generally connected to the output layer to achieve the final output.

In our model, we used *N* as the max-length of the sentences, and short sentences are padded using zeros. The embedding dim is *d*. We took *S*∈ℝ^n*d^ as the input sentence matrix. *W*∈ℝ^n*d^ is taken as the convolution kernel, and *d* is the length of the convolution kernel and is the same size as the dimension of the word embedding. *h* is the width of the convolution kernel. For the input matrix *S*∈ℝ^n*d^, the feature vector $OC=\left(OC_{0},OC_{1},OC_{2},\ldots ,OC_{n-h}\right)\in {\mathbb{R}}^{n-h+1}$ can be obtained by a convolution operation. The formula is as follows:


(1) $$\begin{array}{r} oc_{i}=W\cdot S_{i,i+h-1} \end{array}$$

where *i* = 1, 2, 3, …, *n*−*h*, (·) represents the dot product operation of a matrix. *S*_*i, j*_ is the sub-matrix from row *i* to row *j* of *S*. In CNNs, pooling operations are generally performed after convolution is completed. In the pooling layer, using the maximum pooling operation, we can obtain the maximum value in each filter and extract the most significant features. Zhang et al. (2015) has proved that in various sentence classification tasks, the maximum pooling operation is always better than other pooling strategies in performance. In our model, we also used the 1-max pooling. The main idea is to capture the most important feature *V* corresponding to the specific feature map by selecting the maximum value of the specific feature map. The formula is as follows:


(2) $$\begin{array}{r} V=\max_{0\leq s-h}\left\{oc_{i}\right\} \end{array}$$

As shown in Figure 3, we used different sizes of convolution kernels to extract the features, and then we merged all of the three features into the next layer's input.

### Lexical Rule Level

At this level, the input of the model is the sentimental words of the text. In the work of sentimental lexicon build, we proposed that different combinations of negators, degree words, and simple sentimental words will have different sentimental weights. We used LSTM to extract the lexical rule features.

**Figure 4:**
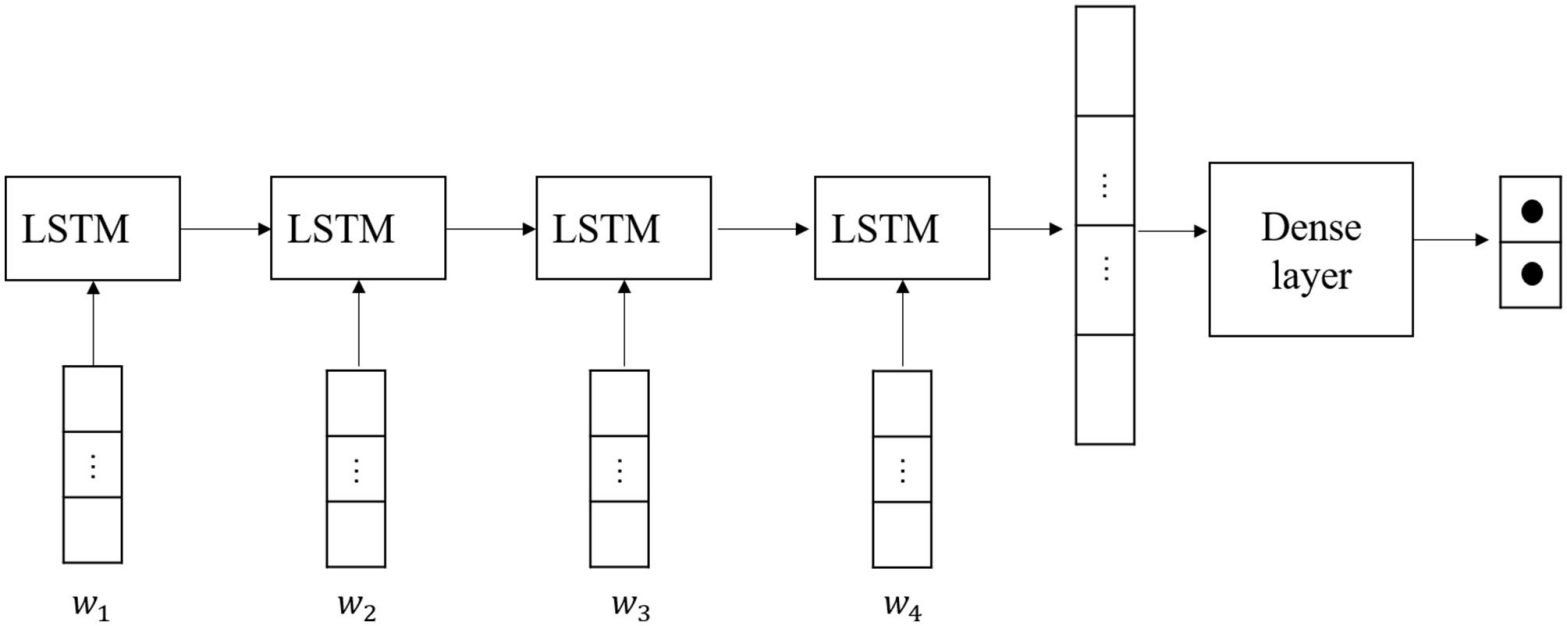
The architecture of long short-term memory (LSTM).

As mentioned in the related work section, RNNs can handle a certain short-term dependency, but if the sequence is long, then the gradient at the back of the sequence is difficult to backpropagate to the previous sequence, so it cannot handle the problem of solving for long-term dependency. In LSTMs, the cell state is introduced and the input is utilized. Different types of gates can save and control information, which can be used to solve the shortcomings of RNNs. So, in this section, we choose LSTMs to replace RNNs. The details of our model are shown in Figure 4.

At each step *t*, the LSTM unit will perform the following actions:

Step1. When the input gate *i*_*t*_ receives the current input *x*_*t*_.

(3) $$\begin{array}{r} i_{t}=\sigma \left(W_{ix}x_{t}+W_{ih}h_{t-1}+b_{i}\right) \end{array}$$

where *h*_*t*−1_ denotes the final hidden state and σ denotes a logistic sigmoid function.
Step2. The cell state at time *t* is $c_{t}^{\sim }$.

(4) $$\begin{array}{r} c_{t}^{\sim }=\text{tanh}\left(W_{cx}x_{t}+W_{ch}h_{t-1}+b_{c}\right) \end{array}$$

If $c_{t}^{\sim }=1$, the current input information can enter the cell state, and if $c_{t}^{\sim }=-1$, the current input information cannot enter the cell state.
Step3. Then, the forget gate value at time *t* is calculated as *f*_*t*_.

(5) $$\begin{array}{r} f_{t}=\;\sigma \left(W_{fx}x_{t}+W_{fh}h_{t-1}+b_{f}\right) \end{array}$$

If *f*_*t*_ = 0, then the information does not pass, and if *f*_*t*_ = 1, then all information is passed to *c*_*t*_ by assigning *c*_*t*−1_ to *c*_*t*_.
Step4. The cell state is then updated to be:

(6) $$\begin{array}{r} c_{t}=\;f_{t}c_{t-1}+i_{t}c_{t}^{\sim } \end{array}$$

Step5. The LSTM is then assigned its final state value:

(7) $$\begin{array}{rcl} o_{t} & = & \sigma \left(W_{ix}x_{t}+W_{ih}h_{t-1}+b_{i}\right) \end{array}$$

(8) $$\begin{array}{rcl} h_{t} & = & \;h_{t}\text{tanh}\left(c_{t}\right) \end{array}$$

### Syntactic Rule Level

According to our research, there are three main syntactic rules in a sentence: structural rules, sentence relation rules, and sentence pattern rules. We referred to these three rules collectively as grammar rules, and the following is the explanation for these three rules.

As for structural rules, we proposed the layer hierarchy for sentimental analysis before (Zhang et al., 2022), including chapters, complex sentences, single sentences, complex sentimental words, simple sentimental words, and characters. Since the sentimental analysis task is mainly in the sentence dimension, the structural rules in this study are only referred to complex sentences and single sentences. A complex sentence is composed of multiple single sentences. The sentimental weight of the complex sentence is accumulated by the sentimental weight of the single sentences according to some rules. The sentimental weight of a single sentence is its weight. Therefore, first, a sentence is marked as a single sentence or a complex sentence according to the punctuation in the sentence. If it is a complex sentence, it is mapped to the vector space according to the number of single sentences.

Sentence relation rules: there are mainly four types of inter-sentence relationship rules, namely, transition relationship, progressive relationship, causal relationship, and hypothetical relationship. In the transition relationship, according to the different transition words, a transition sentence can be divided into one sentence before the transition and another sentence after the transition. The sentimental polarity of the sentences before and after the transition is opposite, and the emotion of the latter sentence is emphasized; in the progressive relationship, the emotion of the preceding and following progressive sentences is gradually strengthened, and more emphasis is placed on the emotion of the latter progressive sentence; in the causal relationship, the emotion of the cause is more emphasized; in the hypothetical relationship, more emphasis is placed on the condition, which weakens the emotion of the latter hypothetical sentence. Based on this, we mapped the turning words in every single sentence to the vector as input. Sentence pattern rules: according to the difference in ending punctuation, it is mainly divided into a declarative sentence, exclamation sentence, and interrogative sentence; among them, the sentence ending with the full stop is the declarative sentence, and the sentimental value of the sentence remains unchanged; the sentence ending with the exclamation mark is the exclamation sentence, and the emotional value of the sentence is enhanced; question sentences can be divided into antonym question sentences and question sentences according to whether they are antonym question words. Question sentences express no emotion, and antonym question sentences emphasize the reverse emotion of the sentence. Therefore, according to the ending symbol, the sentence pattern can be mapped into the vector space.

We can see that the three grammar rules all come from the sentimental analysis method based on the sentimental dictionary, and all influence the sentimental weight of the sentence. But, the existing deep earning models only focus on the text features, and some grammar ruler information will be ignored or changed when using a word embedding to map the text to the vector space and use a neural network model to extract features. This will lead to the lack of processing for some features, thereby reducing the accuracy of the model. We created the grammar vector (GV) feature with the Pseudo-code 1.

**Pseudo-code 1:** Framework of ensemble learning for our system.

**Input:** Sentence, *S*_*i*_; Maximum number of single sentences, *MnS*;
**Output:** Grammar rule vector, *GV*;
Initialize *GV* = [], *GVsS* = [] *GVsS* is the sentence struct feature vector;
*Sentence*_*list*_ = *Sentence segmentation*(*Si*);
**if** *length* (*Sentence*_*list*_) ≤ *MnS* **then**
*GVsS* = [1] * *MnS*;
**else** *GVsS* = [1] * *length*(*Sentence*_*list*_)+[0] * [*MnS*−*length*(*Sentence*_*list*_)];
**end if**
**for** each *simple*_*sentence*_ ∈*Sentence*_*list*_ **do**
Initialize *GV*_*R*_ = [0] * 8
**if** the relation word in *simple*_*sentence*_ **then**
Rwp = find relation word position (relation word)
*GV*_*R*_[*Rwp*] = 1
**end if**
Initialize *GV*_*P*_ = [0] * 4
according to the type of punction in the simple sentence, set the corresponding position of *GV*_*P*_ to 1.
*GV* = *GV*+*GV*_*R*_+*GV*_*P*_
**end for**
*GV* = *GVsS*+*GV*

This model is a combination of three models, and the input of the LSTM and fully connected dense layer need to do some extra processing. Before we used a dataset to do training, we built the sentimental word lexicon for the datasets so that it could be used with this algorithm. Then, we used word2vec to do word embedding. The multi-CNN layer uses the vector-matrix of the sentence as direct input. The LSTM just uses the vector-matrix of the sentimental words contained in the sentence as input. For the fully connected dense layer, we used a grammar vector as the input.

## Experiment

### Dataset and Parameter Setting

We evaluated our model on four datasets: the Tan Songbo hotel review dataset, the NLPCC2014 sentimental analysis task data set, the Douban review dataset (which was crawled by ourselves), and the Weibo comments data set. There are 5,000 positive comments and 5,000 negative comments in each hotel dataset and NLPCC2014 dataset. In addition, there are 50,000 positive comments and 50,000 negative comments in Douban and Weibo data sets, respectively (Fan and Li, 2021). Table 1 shows the statistics of the datasets. The parameters are settled as detailed in Table 2.

**Table 1:** The statics of the datasets.

**Dataset**	**Total number of comments**	**Maximum length of comments**	**Minimum length of sentences**	**Average number of simple sentences contained in each complex sentence**	**Total number of complex sentences**
Douban	100,000	99	11	4.32	90,334
Hotel	10,000	1,985	4	8.74	9,861
nlpcc2014	10,000	1,004	3	5.48	9,258
weibo	119,988	260	1	4.23	100,146

**Table 2:** The parameter setting.

Maxlength of CNN and NN	300
Maxlength of LSTM	200
Embedding dim	300
Embedding method	Word2vec
CNN kernel size	2,3,4
The number of convolutional filters	150
CNN activation	Relu
LSTM units	128
Optimizer	Adadelta

### Ablation Experiments

To evaluate the contribution of different parts of this model, we removed various models and performed experiments. We choose accuracy (Acc) as the evaluation indicator.

As shown in Table 3, the models that include S_LSTM in their names indicate that the input of that LSTM only includes sentimental words, and models that include NN in their names mean that the grammar ruler features were used as input. We can see that, after adding a dense NN layer, all models have higher accuracy than without a dense NN layer. This suggests that the syntactic features extracted from the dense NN model can help obtain more emotional information, thereby improving the accuracy of the model. The results of the MoLeSy and MCNN_S_LSTM demonstrate that using sentimental words input plays a very important role in the inference of the sentiment of sentences.

**Table 3:** The results of ablation experiments.

	**Hotel**	**Douban**	**NLPCC**
MoLeSy	**91.70**	**85.19**	**76.70**
MCNN_LSTM_NN	91.60	84.55	75.49
MCNN_S_LSTM	91.45	84.61	75.20
MCNN_LSTM	91.00	84.30	74.20
MCNN_NN	90.50	84.50	72.50
S_LSTM_NN	90.50	83.94	72.90
MCNN	90.35	83.55	71.25
S_LSTM	89.90	83.20	73.50
LSTM	88.04	83.02	71.55

*Bold values are the maximum number in each column*.

### Comparative Experiment

We used Keras and Python version 2.7 to finish all models, and we trained those models on four GTX 1080 Ti with CUDA. We choose Acc, precision (P), recall (R), and F1 as the evaluation indicators, and we used all four datasets. We choose CNN, LSTM, multichannel CNN and LSTM (MCNNALSTM) (Wang et al., 2016c), SLCABG (Yang et al., 2020), ATTConv RNN-rand (Usama et al., 2020), ABCDM (Basiri et al., 2021), SAMF-BILSTM (Li et al., 2020a), CNN-BiLSTM (sentimental word padding) (Li et al., 2020b), and MC-AttCNN-AttBiGRU (Cheng et al., 2020) as baseline.

The results are shown in Tables 4, 5. It can be seen from Table 4 that the accuracy and the F1 value of our model are highest in all four datasets. This indicates that the use of grammar rules can help get more sentimental features and improve the result of the sentimental analysis. In addition, we found that many models take attention as an important part to extract the feature, which may be better when applied to the extraction of syntactic rules so that no feature extractor is needed. This will possibly be the focus of future research.

**Table 4:** The accuracy of different models on the Hotel and Douban.

	**Hotel**				**Douban**			
	**P**	**R**	**F1**	**ACC**	**P**	**R**	**F1**	**ACC**
CNN	88.18	89.40	88.78	88.61	82.61	86.17	84.36	83.94
LSTM	87.79	89.70	88.74	88.40	86.01	80.54	83.23	83.64
MCNNALSTM	90.61	90.79	90.70	90.52	85.38	84.16	84.77	84.16
SLCABG	91.35	88.91	90.12	90.15	86.13	83.89	85.00	85.11
ATTConv-RNN-rand	92.45	88.42	90.43	90.55	86.19	84.85	85.51	85.55
ABCDM	89.48	92.67	91.05	90.80	84.20	**86.91**	85.53	85.22
SAMF_BiLSTM	**93.25**	87.52	90.30	90.50	84.72	85.49	85.10	84.96
CNN-BiLSTM(SP)	92.32	88.12	90.17	90.30	86.13	83.89	84.77	84.79
MC-AttCNN-ArrBiGRU	91.35	90.99	91.17	91.10	85.53	86.80	86.16	85.99
MoLeSy	90.74	**93.08**	**91.87**	**91.72**	**88.27**	84.17	**86.17**	**86.42**

*Bold values are the maximum number in each column*.

**Table 5:** The accuracy of different models on the Hotel and Douban.

	**NLPCC**				**Weibo**			
	**P**	**R**	**F1**	**ACC**	**P**	**R**	**F1**	**ACC**
CNN	78.32	67.67	72.57	73.56	91.26	96.85	93.97	93.77
LSTM	75.92	68.45	72.00	72.65	92.90	95.99	94.42	94.31
MCNNALSTM	74.36	75.50	74.93	73.80	94.03	95.66	94.84	94.78
SLCABG	79.08	71.46	75.08	75.40	96.19	96.96	96.57	96.57
ATTConv-RNN-rand	**81.70**	67.60	73.98	75.35	95.10	97.11	96.10	96.06
ABCDM	78.59	73.96	76.20	76.05	97.42	96.82	97.12	97.13
SAMF_BiLSTM	74.82	**78.20**	76.47	75.05	94.84	97.15	95.98	95.94
CNN-BiLSTM(SP)	78.32	71.07	74.52	74.80	94.04	**97.31**	95.65	95.58
MC-AttCNN-ArrBiGRU	75.99	78.11	77.03	75.85	98.30	96.79	97.54	97.56
MoLeSy	77.56	77.34	**77.45**	**76.65**	**99.72**	96.73	**98.20**	**98.23**

*Bold values are the maximum number in each column*.

### The Influence of Different Kernel Sizes

**Figure 5:**
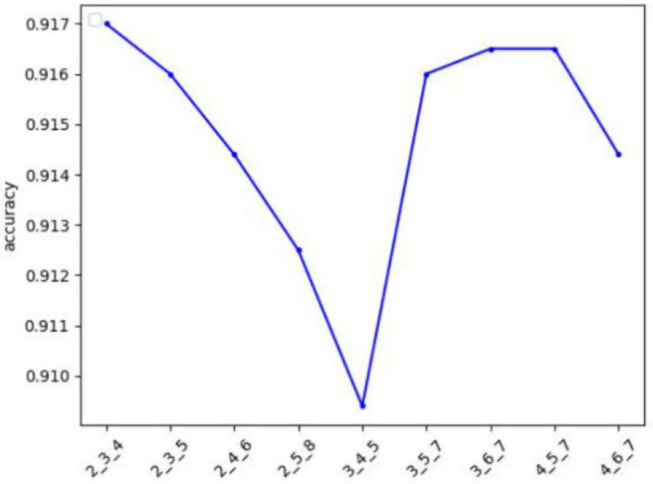
The influence of different kernel sizes on the Hotel data set.

To prove the influence of different sizes of convolution kernels on the experimental results, we conducted experiments on the Hotel data set. We controlled the size of the three convolution kernels to change, and other parameters remained unchanged, as shown in Figure 5, where the *y*-axis is the accuracy rate, and the *x*-axis is the size of the three convolution kernels. It can be seen that when the convolution kernel size is 2, 3, and 4, the accuracy is the highest. This is because, in Chinese, the two-character, three-character, and four-character words are the most used words, so the convolution kernel can extract the most morphological information.

### Accuracy of LSTM-Based Models at Different Epochs

**Figure 6:**
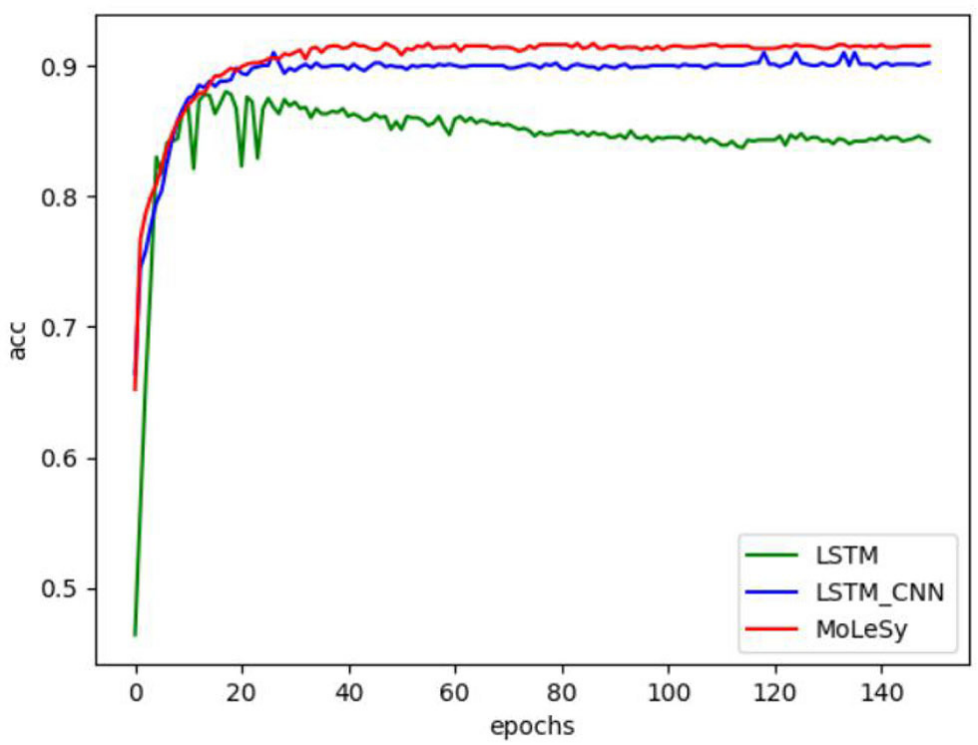
The accuracy at different epochs on the Hotel data set.

To study the accuracy of LSTM-based models in different epochs of training, we conducted experiments on the Hotel dataset, and the experimental results are shown in Figure 6, where epochs = 150, and it can be seen that the accuracy of LSTM_CNN and MoLeSy starts to stabilize at 40 epochs. The accuracy of LSTM fluctuated greatly before 40 epochs and began to stabilize in the later epochs.

### Time Consumption

**Figure 7:**
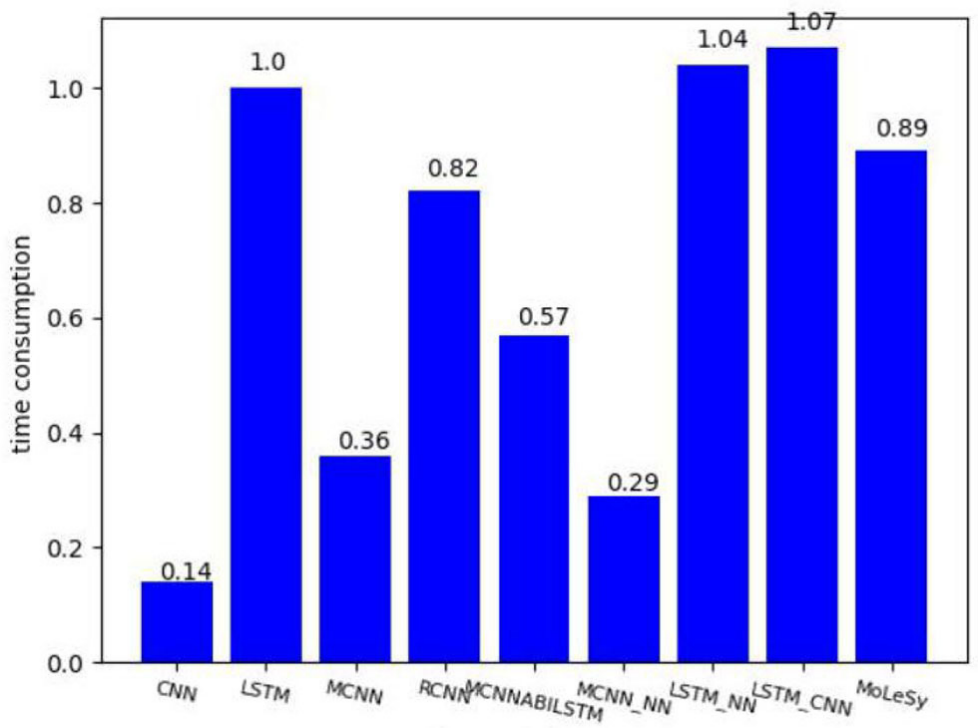
The time consumption based on the LSTM model.

We also compared the time consumption of different models, took the LSTM model as the baseline, and the result is shown in Figure 7.

As we can see from Figure 7, the LSTM-based models need more time-consuming than the CNN-based model. MoLesy is a little faster than the LSTM model, and the reason is that the input of the LSTM layer in the MoLeSy model only contains sentimental words, which is shorter than the length of the whole sentences.

## Conclusion

In this paper, we proposed MoLeSy, an augmented neural network for sentimental analysis, based on the study of Chinese grammar rules and neural network models commonly used in sentimental analysis tasks. The multichannel CNN, LSTM, fully connected dense neural networks, and input of different data for each model together obtain more comprehensive text features. At the same time, this paper proposes and constructs a syntactic rule extractor, which can extract syntactic rules from text and map them to vector space. Comparative experiments with other models on the Tan Songbo hotel data set, NLPCC2014 sentimental analysis task data set, Douban review data set, and Weibo data set showed that the model proposed in this paper can achieve the best performance over previous state-of-art models. Additionally, the model used in the experiments presented in this paper demonstrates that using the input text features as input while adding sentimental word features and grammatical features can achieve more accurate sentimental features. These results suggest a new methodology for Chinese sentimental analysis. This is the first time that all the rules in the method based on the sentimental lexicon for sentimental analysis have been used in the neural network model. The result of ablation experiments showed that the use of the grammar rule feature extraction model can help ascertain more sentimental information and increase sentimental features and final accuracy.

Our model can still maintain the highest accuracy with a faster processor speed than LSTM-based models, which proved that MoLeSy is suitable for the robot to understand human sentiment. We think this study will be helpful in human-robot interaction.

Our model is not without limitation, though: the method of extracting grammar rules is not spectacularly efficient, as only a small set of rules are encoded into the grammar vector, and may ignore some grammar rules as a result. Thus, in our future study, we intended on improving the extraction of grammar features, as we suspected that emphasizing this will reduce the proportion of text features lost. This may require many experiments to prove.

## Data Availability Statement

The original contributions presented in the study are included in the article/supplementary material, further inquiries can be directed to the corresponding author.

## Author Contributions

BZ: conceptualization, methodology, data curation, software, and writing—original draft. HZ: funding acquisition and project administration. JS: writing—review and editing. JC: formal analysis. All authors contributed to the article and approved the submitted version.

## Funding

This work was supported by Fundamental Strengthening Program Technology Field Fund (2021-JCJQ-JJ-0059) and Natural Science Fund of Beijing (4212026).

## Conflict of Interest

The authors declare that the research was conducted in the absence of any commercial or financial relationships that could be construed as a potential conflict of interest.

## Publisher's Note

All claims expressed in this article are solely those of the authors and do not necessarily represent those of their affiliated organizations, or those of the publisher, the editors and the reviewers. Any product that may be evaluated in this article, or claim that may be made by its manufacturer, is not guaranteed or endorsed by the publisher.
